# Reviewer acknowledgement 2012

**DOI:** 10.1186/1751-0759-7-3

**Published:** 2013-02-22

**Authors:** Koji Tsuboi

**Affiliations:** 1 Editor-in-Chief BioPsychoSocial Medicine

## Abstract

The editors of BioPsychoSocial Medicine would like to thank all our reviewers who have contributed to the journal in Volume 6 (2012).

## 

Akihiro Asakawa

Japan

Takanobu Baba

Japan

Adriano Chio'

Italy

Esther Crawley

United Kingdom

Paul Davenport

United States of America

Hans-Christian Deter

Germany

Daniel Devcich

New Zealand

Atsushi Fukao

Japan

Mikihiko Fukunaga

Japan

Hans Grabe

Germany

Harald Gündel

Germany

Masahiro Hashizume

Japan

Junichiro Hayano

Japan

Takahiro Higashi

Japan

Samuel M.Y. Ho

Hong Kong

Reiko Hori

Japan

Yoko Hoshi

Japan

Masako Hosoi

Japan

Hiroshi Kaneko

Japan

Mehran Karimi

Iran

Toshihiko Katafuchi

Japan

Gen Komaki

Japan

Dietrich Lehmann

Switzerland

Sarah Lloyd-Fox

United Kingdom

Mark Lumley

United States of America

Yoshiya Moriguchi

Japan

Emi Morita

Japan

Narumi Nagai

Japan

Shinichiro Nagamitsu

Japan

Jun Nagano

Japan

Yoshikatsu Nakai

Japan

Mutsuhiro Nakao

Japan

Kristy Nielson

United States of America

Shinobu Nomura

Japan

Carl Erik Nord

Sweden

Takakazu Oka

Japan

Isa Okajima

Japan

Toshikatsu Okumura

Japan

Piero Porcelli

Italy

Judith Rabkin

United States of America

Peter Rowe

United States of America

Julie Schirmer

United States of America

Tomotaka Shoji

Japan

Judith Sluiter

Netherlands

Nobuyuki Sudo

Japan

Thomas Suslow

Germany

Yuriko Suzuki

Japan

Masaaki Tanaka

Japan

Mitsunao Tomioka

Japan

Michael Witthöft

Germany

Jong Min Woo

Korea, South

Blake Woodside

Canada

Hidenori Yamasue

Japan

Kazufumi Yoshihara

Japan

Stephan Zipfel

Germany

